# Passive air sampling detects environmental DNA transfer from water into air

**DOI:** 10.1038/s41598-025-26293-6

**Published:** 2025-11-26

**Authors:** Yin Cheong Aden Ip, Gledis Guri, Elizabeth Andruszkiewicz Allan, Ryan P. Kelly

**Affiliations:** School of Marine and Environmental Affairs, University of Washington, Seattle, WA USA

**Keywords:** Environmental DNA, Air eDNA, Passive air filtration, Aquatic biomonitoring, Aerosolization, Evaporation, Non-invasive sampling, Quantitative eDNA analysis, Salmon, Environmental genomics, Ecology, Ecology, Environmental sciences, Ocean sciences

## Abstract

Water and air are generally treated as separate reservoirs of environmental DNA (eDNA) derived from the species resident in those respective environmental compartments. However, it is likely that eDNA routinely crosses the air–water boundary in both directions as a result of deposition, evaporation, or other processes. Here, we systematically tested methods of sampling eDNA at the air–water interface, showing for the first time that aquatic life can be consistently detected under standardized field conditions from passive air samples. We deployed four simple air samplers — three different kinds of filters and one open tray of deionized water — alongside paired water samples and visual counts over a six-week peak run of Coho salmon (*Oncorhynchus kisutch*) at a local spawning stream. We then quantified eDNA concentrations in both air and water (air: copies/cm^2^/day; water: copies/L) using quantitative PCR, to estimate (1) the concentration of target eDNA in air vs. water, and (2) the capture performance of each filter type. Passive air collectors captured quantitative airborne eDNA signals that covaried with salmon counts, despite air eDNA concentrations being approximately 25,000 times more dilute than water, although eDNA recovery varied with sampler design and orientation. We show the air–water interface can be a quantifiable source of aquatic genetic information in this system using simple, passive samplers that do not require electricity, making them appealing for biomonitoring in remote or resource-limited settings. This work points the way to using airborne eDNA as a promising pathway for biological information critical to conservation, resource management, and public-health protection.

**Supplementary Information:**

The online version contains supplementary material available at 10.1038/s41598-025-26293-6.

## Introduction

Monitoring aquatic life is fundamental to understanding ecosystem health, guiding conservation efforts, and managing valuable natural resources^1,2^. Over the past decade, environmental DNA (eDNA) has revolutionized biodiversity assessment by detecting extra-organismal DNA – genetic material shed by organisms into their surroundings. With appropriate calibration or models, eDNA can track organismal presence or abundance^3^. Water-based eDNA surveys have proven particularly effective for monitoring endangered species^4^, early detection of invasive species incursions^5^, characterizing community composition^6^, and even providing quantitative assessments for complementing traditional surveys^3,7,8^.

Meanwhile, airborne eDNA research has emerged as a promising frontier, though to date it has focused almost exclusively on terrestrial organisms. Both active and passive air filtration methods have been shown to recover DNA from mammals, birds, insects, and plants under field and enclosed conditions^9–15^. Intriguingly, these same air-sampling techniques sometimes detect aquatic taxa. For example, Tournayre et al.^16^ reported the occurrence of aquatic species in air eDNA samples, but the authors emphasized that the underlying mechanisms responsible for these detections remain unclear, suggesting multiple, non-exclusive scenarios including prey DNA and soil re-suspension. Crossover signals between air and water have not yet been the focus of a study and are often treated as suspected contamination or attributed to feed or piscivores fecal bioaerosols^15,17–19^, rather than as a genuine ecological signal^16^. As a practical matter, aquatic species signals detected in airborne eDNA surveys are often discarded^17–20^, yet these overlooked detections can hint at an untapped source of real genetic material hence ecological inference.

Environmental systems are inherently interconnected^21^, and basic physics implies that aquatic DNA should appear in air^22,23^. Natural physical processes at the air–water interface — such as evaporation, bubble-burst aerosolization from riffles, and biological processes such as splashes, leaping fish, and churning substrates —all provide plausible mechanisms for transferring eDNA from the water into the air^24–27^. Despite parallel advances in aquatic and airborne eDNA research^18–20,28^ the question of whether aquatic genetic material naturally and routinely moves across media into the air has remained untested.

Existing work has characterized the shedding, transport, and decay of environmental DNA^29–32^, indicating that such processes can shape its concentration in the medium. Yet rates are strongly context dependent, varying with hydrodynamic conditions, turbulence, temperature, UV exposure, substrates, and species behavior, hence, generalized predictions are challenging^30,32,33^. Rather than estimating each component in isolation, other studies have treated their combined effect with a single integrated parameter that enables linking observed fish abundance to measured eDNA, providing a practical, system-specific calibration while acknowledging process complexity^3^. Such integrated approaches have been applied to aquatic eDNA (from trawls and water samples^3,34^) and to active airborne eDNA samplers, but never to passive air samplers, and crucially, never to paired air–water measurements that would allow quantification of cross-medium transfer.

We conducted the first targeted investigation of cross-medium water-to-air eDNA transfer, specifically examining whether genetic material from aquatic organisms can be detected in air samples collected above the water surface. Leveraging the behavior of Coho salmon (*Oncorhynchus kisutch*) during their spawning season^25^, we collected and measured eDNA concentrations in paired water and passive air samples over a six-week peak migration period, with visual fish counts from the staff of a fish hatchery. To evaluate the mechanisms of airborne DNA capture and settlement, we deployed four passive air collection methods: vertically oriented gelatin air filters (commonly used in low-flow air filtration systems), polytetrafluoroethylene filters (PTFE; standard in high-flow air applications), and mixed cellulose ester filters (MCE; traditionally employed for water filtration), as well as an open, horizontal tray of deionized water exposed to ambient conditions^18^. These were chosen for their distinct physical properties, contrasting orientations, and different particle capture efficiencies targeting different aerosol types. By comparing detection sensitivity, temporal patterns, and quantitative performance of these four collectors against water eDNA concentrations and visual counts, we tested if airborne eDNA can consistently track real-world aquatic population dynamics and which collector type would track biological signal most appropriately.

Our overarching goal was to test whether airborne eDNA can quantitatively track aquatic organisms under natural field conditions. Specifically, we hypothesized that (1) DNA from spawning salmon would be detectable in passive air samples collected above the water surface, (2) airborne eDNA concentrations would covary with co-located water eDNA and with visual fish counts, and (3) passive sampler types would differ in the fraction of signal captured because of particle-size and deposition dynamics. This study provides a systematic, field-based investigation of water-to-air eDNA transfer in a natural stream, complementing prior airborne eDNA work that has focused on terrestrial taxa, and offers a transferable framework for other regions and study systems, while recognizing that replication across seasons, sites, and taxa will be needed to assess broad applicability.

## Methods

### Field sampling

We conducted this study near Seattle, Washington USA, in Issaquah Creek, a salmon spawning stream, outside of the Issaquah Salmon Hatchery (47.529501°N, 122.039133°W) from 17 October 2024 to 21 November 2024, with six sampling events. Issaquah Creek is a tributary of Lake Sammamish that drains approximately 175 km^2^ of mixed forested and urban watershed in the Cascade foothills, with discharge of 30–50 cfs (0.8–1.4 m $3$/s) during the study period. The creek supports natural runs of Chinook and Coho salmon, with the Issaquah Salmon Hatchery operating at this location since 1936. The study site was in a riparian corridor approximately 3 km upstream from the creek’s mouth at Lake Sammamish, where the watershed transitions from forested headwaters to developed lowland areas.

We sampled at six time points that spanned the entire Coho salmon (*Oncorhynchus kisutch*) run, from the first arrivals in early fall of August through peak abundance around 17 October 2024 and into the tail end of the migration in November. This schedule ensured that our eDNA sampling captured both the lowest and highest levels of fish activity. Within the same sampling period, visual fish counts were performed by the hatchery staff, and salmon escapement data were obtained from the Washington Department of Fish and Wildlife escapement reports (https://wdfw.wa.gov/fishing/management/hatcheries/escapement#2024-weekly). The hatchery operates a fish ladder with gates that are opened periodically by staff, who conduct visual counts of accumulated fish before allowing passage. This controlled system ensures that all Coho salmon in the study area are counted at this single point before entering the hatchery, creating a direct correspondence between visual counts and the fish population generating eDNA signals in our sampling area. Weather conditions varied throughout the sampling period; environmental metadata for each sampling date – precipitation and mean daily river discharge – are provided in (Table. S2).

#### Airborne eDNA sampling

**Fig. 1:**
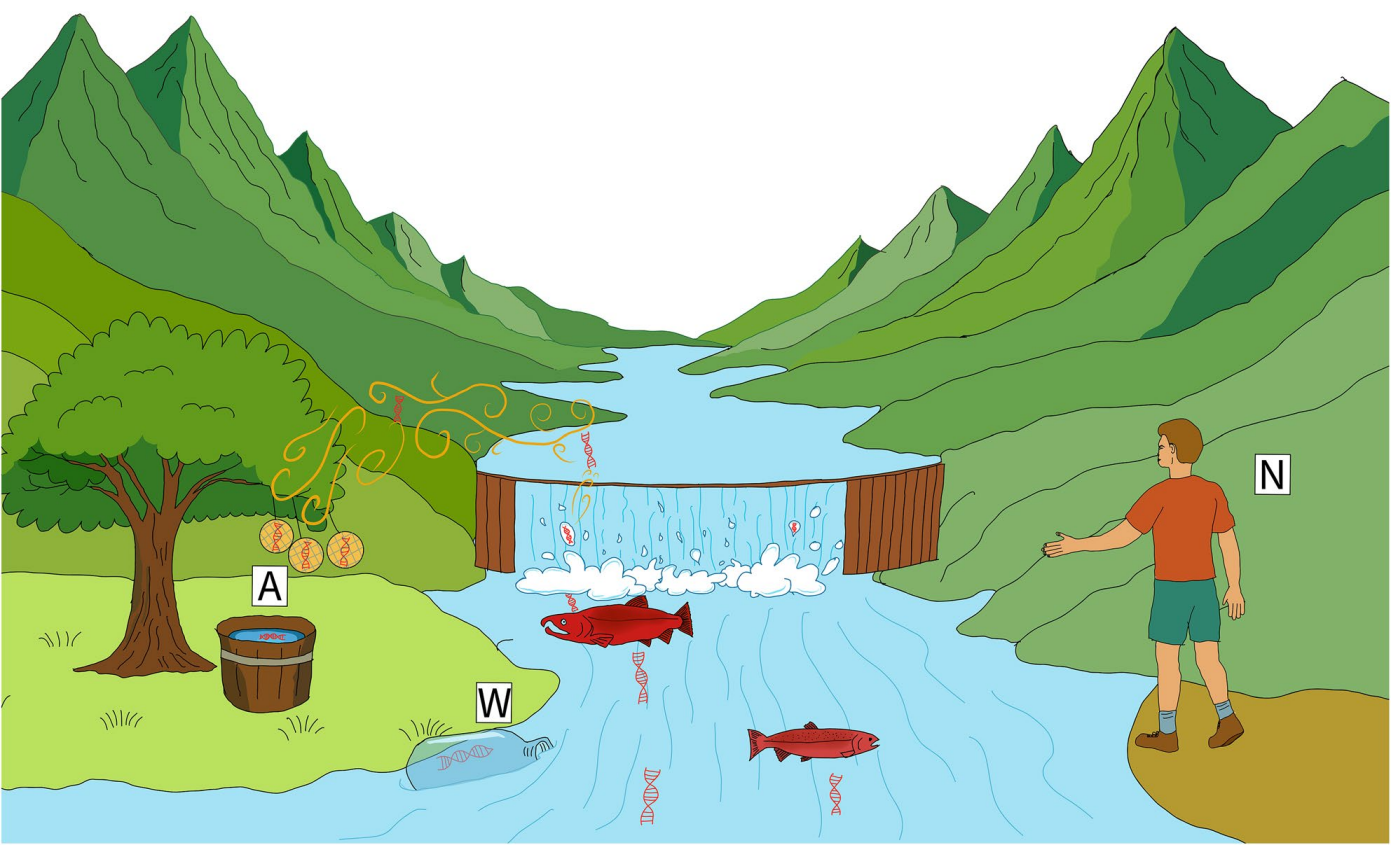
Conceptual illustration of cross‐medium eDNA sampling above a salmon‐spawning stream. Natural processes, including evaporation, bubble-burst aerosolization at riffles and splashes, and the vigorous movement of spawning Coho salmon, launch trace amounts of DNA from the river surface into the atmosphere. Passive airborne samplers, shown here as three vertically hung filters and an open tray of deionized water (A), intercept settling airborne eDNA, while paired water-grab sampling (W) and visual counts by hatchery staff (N) provide concurrent reference measurements.

Sampling for eDNA was conducted in 24-h blocks with deployment and recovery around 9 a.m. (river water was collected only on the first day of each of the six sampling timepoints). Four passive collection methods were evaluated: three filter types – gelatin (Sartorius, 47 mm diameter), PTFE (Whatman, 47 mm diameter), and MCE (Sterlitech, 5.0 $\mu$m pore size, 47 mm diameter) – and an open container of deionized water. The rationale for selecting these materials was as follows: gelatin filters are effective at capturing airborne particles and are particularly suited for applications where maintaining viability (e.g., for subsequent bacterial culturing^35^;) is desired; PTFE filters are noted for their high durability and are widely used in active air sampling experiments^36^; whereas MCE filters, typically employed for water filtration, were included to assess their performance in an airborne context^7^. All three passive filters were deployed via custom 3D-printed “honeycomb” puck filter holders, based on open-source Thingverse designs (IDs 4,306,478 and 979,318; Zachary Gold, pers. comms.), and were suspended from a hatchery railing approximately 3 m above the river water level (Fig. 1), with their collection surfaces oriented vertically. This configuration minimized splash contamination and captures airborne DNA particles driven by gravitational settling. The positioning relative to flowing water demonstrated how these passive collectors operate at a vantage point above the stream, facilitating non-invasive DNA capture in real-world field conditions. Two biological replicates were deployed for both gelatin and PTFE, while only one replicate was used for MCE. After the overnight deployment, filters were carefully recovered using sterile, disposable forceps and immediately immersed in 1.5 mL of DNA/RNA Shield.

The fourth passive collection method was an open container (25 cm width, 30 cm length, 10 cm depth) filled with 2 L of deionized water^18^ also positioned about 3 m above the river and about 30 cm above the suspended passive filters. The open container was also deployed for about 24 h at the same times as the passive filters. The container had an open surface (i.e., oriented horizontally) to capture airborne particles settling by gravity. The surface area of the container was approximately 750 cm^2^, in contrast to the roughly 16 cm^2^ surface area offered by the 47-mm circular filter disks. Only one replicate was used for the open water container method. Note that although 2 L of deionized water was placed in the container, heavy rainfall during overnight deployments sometimes increased the water level. Any debris (e.g., bugs, leaves) was removed from the container before on-site filtration using the same system as for river water samples (see below for details).

#### Water eDNA sampling

At each of six sampling events, we collected one 3 L surface-water grab (3 × 1 L biological replicates) at a fixed site immediately downstream of the fish-ladder gate, adjacent to the Coho salmon aggregation area and concurrent with air deployments. Water was collected in sterile bottles, directly adjacent to the aggregation area. The ladder is operated continuously by hatchery staff throughout the salmon run, ensuring fish passage and visual counts. A total of 3 L of water was filtered on site using a Smith-Root Citizen Science Sampler equipped with 5.0 $\mu$ m mixed cellulose ester (MCE) filters^7^. Three 1 L replicates were processed and immediately preserved in 1.5 mL of DNA/RNA Shield (Zymo Research) using clean disposable plastic forceps. Field-negative filtration blanks (1 L of Milli-Q water each) were also processed using the same system. All equipment was decontaminated with 10% bleach, thoroughly rinsed with deionized water, and handled with gloves to minimize contamination. All filters (passive air, passive open container, water) were stored at –20°C until processing, typically within one week.

The ladder site concentrates upstream migrating Coho salmon and is the control volume where fish are enumerated; sampling water at this location keeps measurements co-located with the airborne collectors and the visual count. One 3 L sample per event provided six time-matched water–air pairs for estimating the water-to-air transfer parameter $\eta$ in the joint model (see Sect. 2.5). In this design, water serves as a calibrated comparator for airborne eDNA^34,37^. This single water sample per event was the paired water measurement used in the joint model.

### Wet-Laboratory procedures

DNA extraction from both river water samples and airborne filter samples was performed using the Qiagen Blood and Tissue Kit according to the manufacturer’s protocols. During extraction, it was noted that the gelatin filters dissolved completely in the DNA/RNA Shield. Samples were vortexed for 1 min, and 500 $\mu$ L of the shield was used for DNA extraction.

qPCR assays targeted a 114-bp fragment of the cytochrome b gene of Coho salmon, using the forward and reverse primers derived from Duda et al.^38^ – COCytb_980-1093 Forward: CCTTGGTGGCGGATATACTTATCTTA and COCytb_980-1093 Reverse: GAACTAGGAAGATGGCGAAGTAGATC. Although Duda et al. validated a TaqMan (probe-based) assay, we used SYBR Green chemistry with the SYBR Select Master Mix (Fisher Scientific) and verified specificity by melt-curve analysis; on an Applied Biosystems QuantStudio 5 real-time PCR system with a 384-well block. Each 10 $\mu$ L reaction consisted of 5 $\mu$ L of SYBR Select Master Mix, 0.4 $\mu$ L of 10 mM forward primer, 0.4 $\mu$ L of 10 mM reverse primer, 2.2 $\mu$ L of molecular grade water, and 2 $\mu$ L of DNA template. Melt curve analysis was performed to confirm the amplification of the target fragment, with an accepted melting temperature of 81 °C ± 1°C. All standards and positives showed a single melt peak at 81 °C ± 1 °C, and no-template and field-negative controls showed no amplification.

Standard curves were constructed using a Coho salmon tissue DNA extract quantified with a Qubit fluorometer (Figure S6). The stock solution was diluted to 1.0 ng/$\mu$ L and designated as 10^6^ copies/$\mu$ L. Serial dilutions were then prepared, with 10^5^, 10^4^ and 10^3^ copies/$\mu$ L run in triplicate, 10^2^ copies/$\mu$ L in quadruplicate, and 10^1^/$\mu$ L copies in triplicate. Assay performance was summarized by the qPCR standard curve and a detection-probability curve derived from replicate standards (Figure S6), and this observation-level uncertainty was propagated in the hierarchical model; accordingly, we reported these performance curves rather than fixed LOD/LOQ thresholds.

### Joint statistical model

#### Visual observation model

In summary, we synthesized three methods of observations (visual counting of fish, water eDNA measurements, and air eDNA measurements) that derived from a single unknown true fish accumulation rate (denoted here as $X$). This joint approach allowed us to estimate the aerosolization factor ($\eta$; the fraction of water eDNA transferred to air), the effectiveness and reliability of different passive air filtering techniques ($\tau$), and the replicability of various filters ($\rho$) throughout the 6-week peak coho salmon spawning period. Accordingly, the water measurement at the fish ladder counting site was used as a time-matched index of local fish density that drove the air compartment in the joint model. At each sampling event, we obtained one water measurement at the ladder site and contemporaneous air measurements from the passive air collectors (by filter type with biological replicates). The aerosolization parameters were estimated from the six paired water–air observations across the study period.

We modeled the upstream migration of Coho salmon (*Oncorhynchus kisutch*) as arising from the inferred unknown true density of fish, *X*. As individuals moved upriver, fish accumulated in a holding area immediately downstream of the hatchery river dam (our water and air sampling location). At discrete times $t$ (six weekly sampling dates between 17 October and 21 November 2024), the hatchery staff opened the ladder gate to allow passage into holding tanks. Between successive gate-opening events ($\Delta t$), additional coho salmon arrive and join the backlog in the holding area, increasing the number of individuals awaiting passage. We denoted the true daily accumulation rate ${X}_{t}$ (also fish density at the river dam) in units of fish/day at time $t$, and we denoted the counting effort as $E$ (measured as the elapsed number of days between consecutive gate openings; ${E}_{t}=\Delta t$; hence ${E}_{t=1}=0$). Here, ${E}_{t}$ (days) represented the count effort, converting the daily accumulation rate ${X}_{t}$ (fish/day) into the expected accumulated count ${\lambda }_{t}={X}_{t}\cdot {E}_{t}$ (fish) in Eq. (1). Counts were full enumerations at the ladder immediately before each opening and hence, person-hours are not modeled separately. Prior to each gate opening (at time $t$), the hatchery staff conducted a visual count ${N}_{t}$ of accumulated fish. Assuming ${X}_{t}$ remained relatively constant between successive gate-opening events ($\Delta t$), we modeled the observed fish counts as a Negative Binomial process:

1 $${\lambda }_{t}={X}_{t}\cdot {E}_{t}$$

2 $${N}_{t}\sim \text{Negative}\hspace{0.25em}\text{Binomial}({\lambda }_{t},\phi )$$

where, $N$ was the visually observed number of fish at time $t$, ${\lambda }_{t}$ was the expected number of fish accumulated over the ${E}_{t}$-day interval with a fixed overdispersion parameter shared across time points ($\phi =20$; hence variance $\lambda +\frac{\lambda^2}{\phi }$)^3,39^. Given the small number of time points (n = 6), the overdispersion parameter $\phi$ was fixed to a literature-supported value rather than estimated.

#### Molecular process model

We denoted $W$ as the unobserved eDNA concentration (copies/L) in the single water sample collected at each time point $t$ and $\omega$ as the “integrated eDNA factor" – the conversion factor between fish accumulation ($X$) and eDNA concentration ($W$; see Guri et al.^3^ for further interpretation of $\omega$). Here, $\omega$ was treated as a population-scale factor that aggregated per-fish eDNA emission (i.e., the average shedding rate per individual), together with decay, transport, and dilution at the site; we assumed it was approximately constant over the 24-h sampling window. Individual-to-individual variation was not modeled explicitly and contributed to residual variance. We express the relation between the fish density and water eDNA concentration as:

3 $${W}_{t}={X}_{t}\cdot \omega$$

We denoted $A$ as the unobserved eDNA concentration (copies/cm^2^/day) in the air at time $t$ which was filtered using passive collection method $j$. We indexed air collection methods as $j\in \{1,\dots ,4\}$ index air-collection methods: 1 = PTFE, 2 = gelatin, 3 = MCE (air-suspended), 4 = DI-water tray. We modeled the air eDNA concentration as a log-linear function of the water eDNA with intercept $\eta$, slope = 1, and error term $\varepsilon$ (unexplained variability; time and filter specific):

4 $$\text{ln}({A}_{tj})={\eta }_{j}+\text{ln}({W}_{t})+{\varepsilon }_{tj}$$

Here, the intercept $\eta$ was be interpreted as the water-to-air transferability (or dilution) factor and $\varepsilon$ as the error term parameter (similar to the sum of squares error) from a linear regression where ${\varepsilon }_{tj}\sim \mathcal{N}(0,{\tau }_{j})$.

For the two filter types with biological replicates ($j\in \{1,2\}$; PTFE and gelatin); we sampled two biological replicates and we used the mean of those biological replicates to determine the average concentration in the air $A$ at time $t$ as following:

5 $${ln}({A}_{tjb})=\text{ln}({A}_{tj})+{\delta }_{tjb} \quad {\text{for}}\hspace{0.25em}j\in \{1,2\}$$

where ${\delta }_{b}$ indicated the deviation of individual biological replicate from the average concentration (${A}_{tj}$), following a normal distribution with mean 0, ${\delta }_{tjb}\sim \mathcal{N}(0,{\rho }_{j})$, with ${\rho }_{j}$ indicating the magnitude of the replicates’ deviation from the mean sample. Because we had only two replicates at each sampled time, we imposed a sum to zero constraint on the replicates ($b$) collected from a single sampling time ($\sum_{b}{\delta }_{tj}=0$).

To estimate the levels of eDNA concentration in water ($W$) and air ($A$), we utilized the qPCR observation models (as described in Guri et al.^37^ and Shelton et al.^34^, with slight modifications). The model compartment utilized the standard curve samples to estimate the intercept ($\phi ,\beta_0,\gamma_0$) and slope ($\beta_1,\gamma_1$) parameters between the known concentration ($K$) and the observed data ($Z$ and $Y$) from qPCR machine as follows:

6 $$Z_{kr} \sim \text{Bernoulli}(\psi_{k})$$

7 $$psi_{k} = 1 - \exp(-K_{k} \cdot \theta)$$

8 $$Y_{kr} \sim \text{Normal}(\mu_{k}, \sigma_{k}) \quad \text{if } Z_{kr} = 1$$

9 $$\mu_{k} = \beta_{0} + \beta_{1p} \cdot \ln(K_{k})$$

10 $$\sigma_{k} = \exp(\gamma_{0} + \gamma_{1} \cdot \ln(K_{k}))$$

where we denoted $Z$ is the binary outcome of target amplification for sample ($k$) and technical replicate ($r$) being present (1) or absent (0) following a Bernoulli distribution given the probability of detection $\psi$ for each sample ($k$). We denoted the parameter $\phi$ as the intercept of the function between probability of detection $\psi$ and the known DNA concentration ($K$; copies/$\mu$ L reaction) as the predictor variable. Additionally, for Eqs. 8–10, $Y$ was the observed cycle threshold (Ct) for sample ($k$) and technical replicate ($r$) which followed a normal distribution with mean $\mu$ (mean Ct) and standard deviation $\sigma$ for each sample ($k$). We modeled $\mu$ as a linear function of known eDNA concentration ($K$) with intercept $\beta_0$ and plate specific ($p$) slope $\beta_1$ and the standard deviation $\sigma$ of the observed Y as an exponential function of known eDNA concentration with intercept $\gamma_0$ and slope $\gamma_1$.

Subsequently, we build the same model compartment for estimating eDNA concentration in water and air by substituting ${U}_{t}$ and ${Q}_{tj}$ (and ${Q}_{tjb}$ for $j\in \{1,2\}$) respectively, with ${K}_{k}$ through Eqs. 6–10 (see Figure S1), where $U$ and $Q$ are concentration normalized per reaction volume ($V$ = 10 $\mu$ L for all reactions including water and air samples), water volume filtered in the field ($F$ = 1 L for all water samples), surface area ($S$ = 16 cm ${}^{2}$ for gelatin, PTFE, and MCE, and 750 cm^2^ for the open containers of deionized water; for air samples only), and for passive deployment time ($P$ = 1 day across all samples at all time $t$; for air samples only) of $W$ and $A$ respectively as follows:

11 $${U}_{t}={W}_{t}\cdot F/V$$

12 $${Q}_{tjb}={A}_{tjb}\cdot {S}_{tj}/V\cdot P$$

The intercept and slope parameters (from Eqs. 7, 9, and 10) between qPCR observations and eDNA concentration of water, air and the standard samples were shared between model compartments.

### Model conditions

The joint model (Figure S1) was implemented using the Stan language as implemented in R (package: Rstan) running four independent MCMC chains using 5000 warm-up and 5000 sampling iterations (for parameters and their prior distributions see Table S1). The posterior predictions were diagnosed using statistics^40^ and considered convergence for values less than 1.05 and effective sample size (ESS) greater than 1000 for all parameters. Additionally, the posterior predictive checks were used with results presented in the Figure S5. Observations from different gate-opening intervals were assumed independent; with n = 6, we did not fit an explicit temporal correlation structure.

## Results

Airborne eDNA was detected across all passive methods and covaried with Coho salmon abundance in the river during the upstream migration period. Detection efficiency and signal strength varied considerably among the different passive airborne eDNA capture methods. We then estimated the water-to-air dilution factor and compared temporal congruence among collectors to identify which designs best tracked the biological signal.

### Fish accumulation based on visual counts and eDNA in river water

**Fig. 2:**
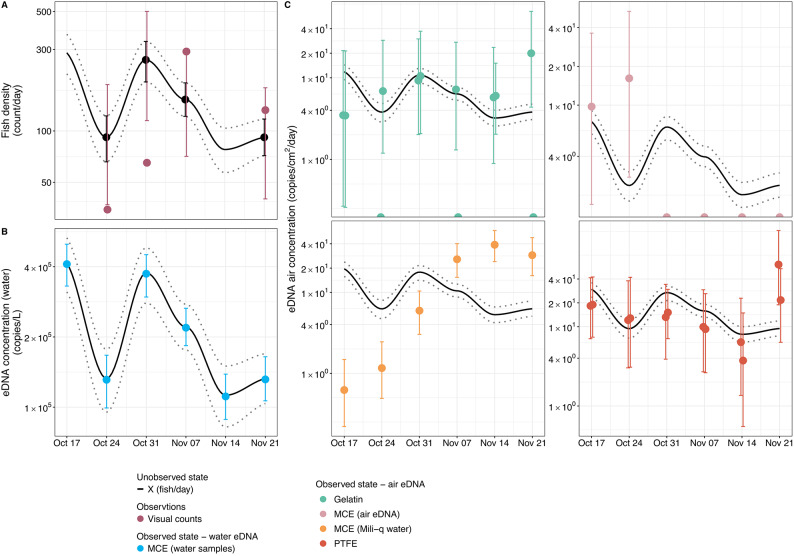
The temporal dynamics of estimated fish density in units of fish/day ($\text{X}$; black dots and smoothed lines with 95% confidence intervals indicated with error bars in panel A and dotted lines throughout) from 17^th^ October to 21^st^ November compared to the posterior distributions (and their 95% confidence intervals indicated by vertical bars) of eDNA concentrations (copies/L) in water (B), and eDNA concentrations (copies/cm^2^/day) in air using various filter types (C). In panel A we also show the observed visual counts (dark rose dots) normalized per counting effort (E) with the 95% posterior probabilities (dark rose bars) derived from the Negative binomial distribution with mean $\text{X}$ (fish density) and the fixed overdispersion parameter ($\upphi$=20). The jitter in x-axis for panel A and for Gelatin and PTFE plots in panel C are for visualization purposes only, i.e., no difference in collection dates.

Coho salmon migration occurs not as a single continuous event, but rather as a series of distinct burst peaks from mid-October through late November, where the peaks are highly likely to be connected with environmental factors such as water temperature and discharge. The average daily accumulation rate ($X$; black line in Fig. 2) was estimated at 160.4 fish/day with peaks exceeding up to 286 fish/day and low activity of ca. 78 fish/day (Fig. 2).

Because both observation methods (river water eDNA and visual observation) are jointly used to estimate the daily accumulation rate ($X$), their concordance was best evaluated through the parameter $\omega$. A converged and narrowly distributed $\omega$ parameter indicates strong agreement between the two methods and simultaneously a reliable conversion parameter from fish/day to eDNA copies/L. In this case, $\omega$ = 9.578 (95% quantile range of 9.352 to 9.804; Figure S4), suggesting consistent concordance between observed fish counts and eDNA concentrations hence, biologically, this implies that an accumulation rate of 1 fish/day corresponds to approximately 15,000 ($\pm$ 3000) copies/L.

### Air eDNA signals

Airborne eDNA originating from Coho salmon was successfully detected across all passive air collection methods deployed (gelatin, PTFE, MCE filters suspended in air, and open containers of deionized water—MCE DI water). Across airborne sampling approaches, airborne eDNA concentrations were approximately 25,000 × lower than co-located water concentrations (ranging $\sim$ 15,000–50,000 × depending on sampler type; Table 1; Figure S2A). These values are summarized by an integrated dilution parameter ($\eta$) defined in Methods (Table 1; Figure. S2A). Despite the general consistency in estimating the water-to-air dilution coefficient, method-specific variations in collection efficiency were observed (Table 1; Figure S2A). PTFE filters exhibited higher capture efficiencies, collecting 2 times more eDNA than the mean across all air sampling methods (Table 1). Deionized water tray (MCE DI water) demonstrated the second highest efficiency (1.3 times the average; Table 1), while gelatin filters and air-suspended MCE filters showed comparatively lower capture efficacy (0.8 and 0.5 times the average, respectively; Table 1).

**Table 1:** Estimated posterior means of dilution parameter ($\upeta$), standard deviation of the residuals ($\uptau$), biological replicability ($\uprho$), and capturing efficiency (${\text{e}}^{(\upeta -\overline{\upeta })}$).

Filter type	**Dilution** (in $lo{g}_{e}$)	**Standard deviation**	**Biological rep.** **error magnitude**	**Capturing efficiency**
	($\eta$)	($\tau$)	($\rho$)	${e}^{(\eta -\overline{\eta })}$
Gelatin	$-10.45$	0.473	0.386	0.785
PTFE	$-9.53$	0.570	0.154	1.979
MCE Air	$-10.91$	0.949	-	0.496
MCE DI water	$-9.95$	1.780	-	1.295

In terms of alignment with the biological activity, PTFE and gelatin filters showed the strongest covariation with the daily fish accumulation patterns, exhibiting the lowest residual error magnitude (expressed as the standard deviation of $\varepsilon$) with $\tau$ = 0.473 and 0.570, respectively (Table 1; Figure S2B). Conversely, MCE DI water, despite having the largest surface area, showed less agreement with the fish migration dynamics ($\tau$ = 1.780; Table 1; Figure S2B). The MCE air suspended filters performed least effectively in tracking temporal migration patterns, failing to amplify coho salmon DNA beyond the first two weeks of the sampling campaign Fig. 2C.

Subsequently, biological replicates for gelatin and PTFE filters revealed additional insights regarding methodological robustness and reproducibility. PTFE filters produced the most consistent quantifications, with lower variance (expressed as the standard deviation of $\delta$) between replicates ($\rho$ = 0.154; Table 1; Figure S2C), whereas gelatin filters showed a higher degree of variability ($\rho$ = 0.386; Table 1; Figure S2C), indicating reduced reproducibility of quantitative outcomes.

In sum, these performance differences across sampling methods likely reflect inherent physical and operational characteristics of each filter type and collection method, which in turn influence their ability to capture either discrete or cumulative biological signals from the source species.

### Model diagnostics

Convergence and reliability of the Bayesian model were assessed through comprehensive diagnostics. All parameters (Table S1) exhibited reliable Gelman-Rubin convergence statistics ($\widehat{R}<1.01$) and effective sample sizes (ESS) exceeding 1000 per parameter, indicating successful convergence and efficient mixing of the four independent chains (Figure S3A). No divergent transitions were detected during sampling, and the maximum tree depth was not exceeded, indicating no issues with divergence or exploration limits (Figure S3B). The posterior likelihood demonstrated convergence before the sampling phase began, with all chains exhibiting high mixing, confirming robust exploration of the parameter space (Figure S3B).

Prior sensitivity analyses revealed that posterior estimates differed from priors, demonstrating that the posteriors were appropriately updated based on the observed data rather than being heavily influenced by prior assumptions (Figure S4). Additionally, the posterior predictive checks (PPC) demonstrated that the model reliably reproduced the observed data, supporting the validity of parameter estimates (Figure S5). Collectively, these diagnostics confirm the reliability and validity of the Bayesian model used here.

## Discussion

### A new dimension of biodiversity monitoring

Our study shows, for the first time, a field-based proof-of-concept that passive airborne eDNA sampling can capture and track molecular signals from aquatic organisms. In this system – Coho salmon in a hatchery-controlled river reach – fully passive airborne eDNA sampling (no active airflow) recovered salmon genetic material and covaried with water eDNA concentrations and visual counts of salmon across six weekly sampling events, providing proof-of-concept in this context that the air and water assays reflected a common underlying phenomenon. This suggests a new and complementary pathway for assessing aquatic biodiversity – through the air, without ever touching the water. In salmon-spawning streams, genetic material from the river is transported into the air likely by evaporation, bubble-burst aerosolization at riffles and splashes, and the rigorous churning of spawning fish^41,42^. Collections from multiple passive samplers, when compared with conventional water-based eDNA assays and daily visual counts, were consistent with a quantitative relationship between airborne eDNA concentration and salmon density.

Detections of aquatic taxa in airborne surveys are often dismissed as contamination^15,17–19^. Our data suggest otherwise. In this system, the airborne salmon eDNA signal is consistent with an ecological origin rather than laboratory or procedural artifact. Additionally, it is likely that environmental conditions such as wind speed, relative humidity, and temperature can determine the spatial and temporal distribution of the aerosolized aquatic eDNA^43,44^. For example, high humidity and rainfall force rapid settling and very localized deposition while dry, windy conditions might carry genetic plumes further distances downwind^45,46^. Although quantifying these effects was beyond the scope of this study, they – together with hydrological variables – should be considered when evaluating how passive samplers capture transient pulses of biological activity.

### How sampler design may shape signal detection

The interpretations below are mechanistic hypotheses consistent with our observed patterns and prior studies; we did not test these mechanisms directly. In concordance with other studies tracking real biological life^47^, our passive filters deployed for 24-h (vertically oriented gelatin and PTFE) acted as higher-resolution “fish-activity” samplers. Such processes can occur due to ambient air currents likely sweeping fine, splash-generated aerosols rich in salmon DNA onto filter membranes, yielding traces of DNA that could rise and fall in sync with live fish counts and water-eDNA levels^48^. In contrast, the large horizontal tray of deionized water seemed to function more like a hydraulic-driven deposition trap. The tray saw a steady accumulation of eDNA over the six weeks and thus could have been collecting more coarse spray, foam, and decay-derived particulates from river turbulence and from decomposing carcasses^42,49^. Because salmon carcasses often remain in shallow banks and backwaters as the spawning season progresses, it is plausible that river turbulence and discharge could generate larger droplets over decomposing tissue, potentially facilitating eDNA dispersal^41,50^. Although to our knowledge there is no study that has investigated this process in particular, we hypothesize that the coarse droplets settle rapidly and dominate deposition on horizontal collectors while the fine fraction produced by active fish movement could be more volatile and under-represented. This could explain the more discrete nature of signals recovered from gelatin and PTFE filters vs. the more cumulative signal of the water tray (which may integrate both flow-driven and decay-driven processes into a steadily rising accumulation curve). While carcass-derived particulates are a plausible contributor to the cumulative signal observed in the horizontal tray, we did not directly quantify the relative contributions of carcass material and live shedding. Disentangling these sources will require targeted experiments that track release from live fish and from decomposing tissue under controlled conditions.

The filter materials and operational context also shaped the air sampling performance. PTFE filters, known for their durability, delivered the most consistent results of all the passive filtration methods; gelatin filters yielded the highest sensitivity but showed greater variability; mixed cellulose ester filters captured negligible airborne DNA; and the open tray, with roughly 50 times more surface area than the vertical filters, recovered the highest total DNA yield. These samplers can respond differently to weather primarily due to their material composition. In the physical form, through our visual observations when handling the air samples, PTFE filters are generally unaffected by rain (always intact when retrieved), whereas gelatin filters typically dissolve when wet. Housing is another tunable design variable^13^: enclosed or rain-shielded filter holders may mitigate wetting and UV exposure while maintaining deposition. Our deployments used open holders, which likely contributed to wetting of gelatin filters during rain events. Additionally, the environmental variables might introduce some untestable trade-offs. For example, heavy rain can dilute the accumulated eDNA but may also scour additional airborne or splash-borne DNA into the water. Real-world debris, such as leaves, insects, sediment, can also wash into the tray, increasing the risk of clogging or necessitating pre-filtration before DNA extraction. Rain and wind can also deliver non-local DNA to open trays; while the time-matched concordance with co-located water eDNA and visual counts (Fig. 2) argues for a predominantly local signal in this study, we cannot exclude external inputs. Although our sample size is too small to quantify these opposing effects, future work should explicitly test how precipitation intensity influences both concentration and total yield^12^. Targeted experiments with co-measured meteorology and particle data will be required to test these mechanisms (see Discussion 4.4).

### Airborne eDNA as a fraction of waterborne eDNA

Across airborne sampling approaches, airborne eDNA concentrations were approximately 25,000 × lower than co-located water concentrations, with values by sampler spanning roughly 15,000–50,000 ×, at levels approaching the lower limits of qPCR detection. Informally, the air compartment can be viewed as a highly diluted reflection of the water signal at this site, but we emphasize this is a descriptive metaphor, not a tested mechanism. Despite this extreme dilution, our rigorous sampling, laboratory methods, and statistical models reliably picked up those sparse molecules, obtaining consistent, quantitative signals across the entire spawning season. To put this in perspective, dissolving a teaspoon of salt into a large aquarium yields a similar dilution magnitude: only a tiny fraction of DNA shed into the water ever makes it into the overlying air, yet those few copies suffice to track real-time salmon activity. In particular, vertically oriented filters intercepted transient eDNA peaks that rose and fell broadly in concert with visual counts, suggesting that even at extreme dilution, airborne eDNA can still capture fine scale changes in fish presence that reflect real-world population dynamics.

Longer passive‐sampler deployments naturally accumulate more settled eDNA, but they also expose collected material to ultraviolet radiation, microbial degradation and fluctuating humidity, all of which erode DNA integrity over time^33^. Hinds et al.^29^ showed that waterborne eDNA degrades with a half‐life of hours to days under natural sunlight and microbial loads, suggesting that once deposited on the filter, airborne fragments may decay on comparable or even faster timescales. By contrast, Klepke et al.^18^ found that passive air particle collections continued to accrue new species detections for up to 96 h, without specifying when deposition begins to be outpaced by degradation. In practical terms, this could mean that in a longer deployment much of the DNA captured from early days may degrade before retrieval which potentially can skew the majority of signal by reflecting material deposited in the final timespan of sampling. Here, we show that selecting a 24‐hour deployment is sufficient for capturing biological signals which might alleviate the potential limiting factor of post‐deposition loss^12^. However, future work should explore these plausible interpretations by varying deployment lengths from a few hours to several days, and pairing them with controlled decay assays, for example by spiking synthetic DNA onto filters and tracking its persistence, to determine the point at which accumulation and degradation balance.

Our findings also agree with the recent work by Jager et al.^47^ who showed that passive air samplers outperform active pumps by sampling intermittent, DNA-rich plumes over long intervals and detecting greater species richness. In our streams, vertical filters captured transient bursts of salmon eDNA in near real-time, while the open tray, and likely longer deployments, would tend to smooth those peaks into an integrated signal. Contrastingly, active-pump systems operating for only hours would average across plumes and risk overlooking fine-scale temporal changes.

By systematically mapping this interplay between deposition and degradation, and by benchmarking passive against active approaches, we can establish best‐practice guidelines for airborne eDNA sampling durations. This effort would mirror how water‐based eDNA workflows define optimal filtration volumes and storage times^20,51^ and would enable robust, context‐specific deployments that maximize genuine signal recovery for real‐time biodiversity monitoring. Airborne eDNA decay dynamics remain largely unquantified. While aquatic decay studies provide context, degradation in air likely differs due to UV exposure, desiccation, and distinct microbial communities; direct measurements across environmental conditions are a clear priority.

### Minimal tools, maximum reach and navigating limitations

Perhaps the most striking result from our work is the simplicity and versatility of passive airborne sampling. No pumps or power are needed, and equipment is extremely low cost and easy to deploy. This minimal-infrastructure approach makes it viable for remote headwaters, steep mountain channels, urban stormwater networks, and contaminated waters where sampling is unsafe^30,52^. Despite this being the first study to explore the link between water-to-air eDNA migration, this could open opportunities for future monitoring scenarios where access to water is limited (e.g. droughts, floods and public-health risks such as bacterial outbreaks in stagnant waters). Airborne eDNA may offer resilient pathways for rapid invasive-species alerts, real-time disease surveillance in flood-prone wetlands and non-invasive population censuses in protected spawning grounds.

However, air-sampling replication was imbalanced, with two biological replicates for gelatin and PTFE filters and one for MCE filters and the deionized-water tray. This imbalance limits the strength of cross-method contrasts, so future deployments should harmonize replication across sampler types. Our temporal replication comprised six co-located sampling dates during the peak spawning period; while adequate for estimating a system-specific dilution magnitude, this sample size limits inference about temporal drivers and broader applicability. Additionally, our focus on peak spawning, when salmon exhibit maximal surface activity, means that applicability to other seasons, less surface-active species, or lentic systems requires further investigation.

Naturally, challenges remain, as passive deployments rely on surface-area-by-time metrics rather than standardized air-volume units, complicating direct comparisons across studies. Optimal exposure times must balance accumulation against DNA degradation from UV, microbes and moisture^33^. Weather variability in wind, humidity and rain can alter deposition rates and sampler efficiency^12,28^. In cold climates, freezing of exposed holders can alter particle deposition and DNA retention, and thaw may physically strip accumulated material; enclosed/rain-shielded housings and shorter deployments could mitigate this, but dedicated cold-weather tests are needed. Further studies should shed light on environmental conditions (i.e., temperature, humidity, seasons, etc.,) refine sampler design, systematically compare vertical and horizontal orientations, explore automated or drone-based retrieval and integrate river discharge and meteorological data into quantitative models^31,32,41,45^. In particular, upwind/downwind replicate samplers with co-measured wind direction/speed and precipitation would help test signal provenance. Because we had relatively few time points, the Negative Binomial overdispersion ($\phi$) parameter was fixed to a literature-supported value and observations from different gate-opening intervals were treated as independent; with higher-frequency sampling, $\phi$ could be estimated from the data and an explicit temporal autocorrelation term incorporated. Relatedly, our inference treats per-capita shedding and decay as aggregated into $\omega$ and approximately constant over each 24-h deployment; individual heterogeneity is absorbed into the residual variance, and future work with size data or mechanistic submodels could relax this assumption. This study evaluates a single stream/hatchery reach and species; replication across systems and taxa will be required to assess generality.

Our study begins to chart a portion of airborne eDNA ecology in five key dimensions^28^: 1) we support a local origin by matching airborne DNA trends to co-occurring water eDNA and fish counts, 2) we highlight likely transport mechanisms such as evaporation, bubble bursts, and fish activity, 3) we quantify dispersal and dilution across the water–air boundary, 4) we demonstrate fate through differential deposition on vertical filters and horizontal trays, and finally, 5) we show that airborne DNA fragments remain amplifiable, offering an initial glimpse into their molecular state after transport. The potential underlying processes suggest that air could act as a dilute, but still informative, extension of the aquatic environment, representing biological signals that are real, quantifiable, and ecologically meaningful. These insights lay the groundwork for future studies on persistence, degradation and particle-size distributions from airborne eDNA^53^.

## Supplementary Information

Supplementary Information.

## Data Availability

The authors declare that they have no competing interests. All data needed to evaluate the conclusions in this paper are available in the main text and/or the Supplementary Materials. Raw data, R and Stan model codes are available on https://github.com/gledguri/Air-eDNA-quant. Additional data, code, and materials will be made available upon reasonable request. No materials were subject to material transfer agreements (MTAs).
