# Supplementary material for: Passive air sampling detects environmental DNA transfer from water into air
Source: Sci Rep. 2025 Nov 26;15:42245. doi: 10.1038/s41598-025-26293-6 (PMC12657985; doi:10.1038/s41598-025-26293-6)
Supplement: Supplementary file 1 — Supplementary Information. [file 41598_2025_26293_MOESM1_ESM.pdf]

Supplementary Material

Passive air sampling detects environmental DNA transfer from water into air

Yin Cheong Aden Ip[1]* Gledis Guri[1]* Elizabeth Andruszkiewicz Allan[1]
Ryan P. Kelly[1]

October 24, 2025

1 School of Marine and Environmental Affairs, University of Washington, Seattle, Washington, USA
* shared first authorship

corresponding author **adenip@uw.edu**

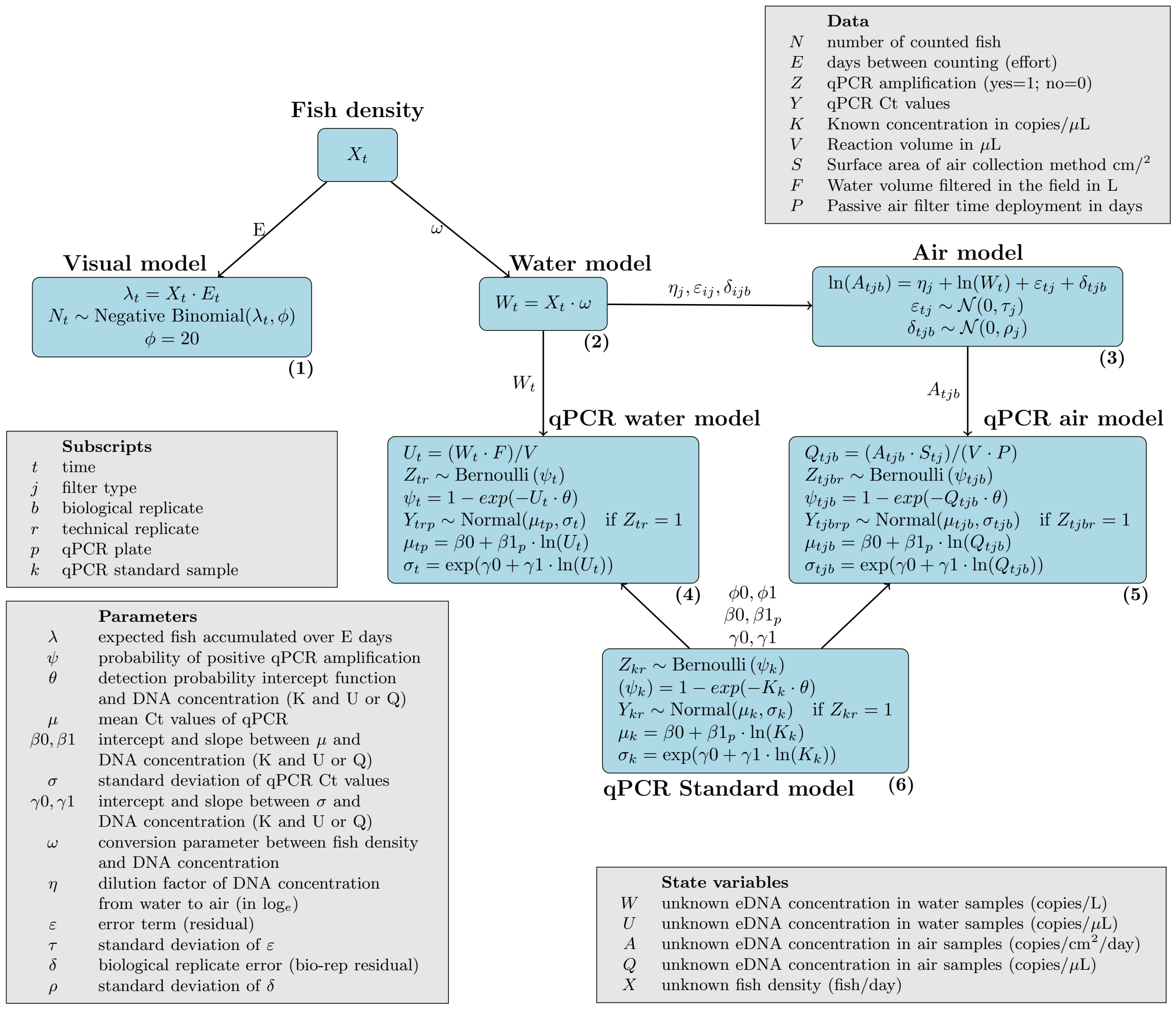

Figure S1: Directed acyclic graph (DAG) of joint Bayesian model for linking salmon migration dynamics from visual observation, water and air eDNA concentration.

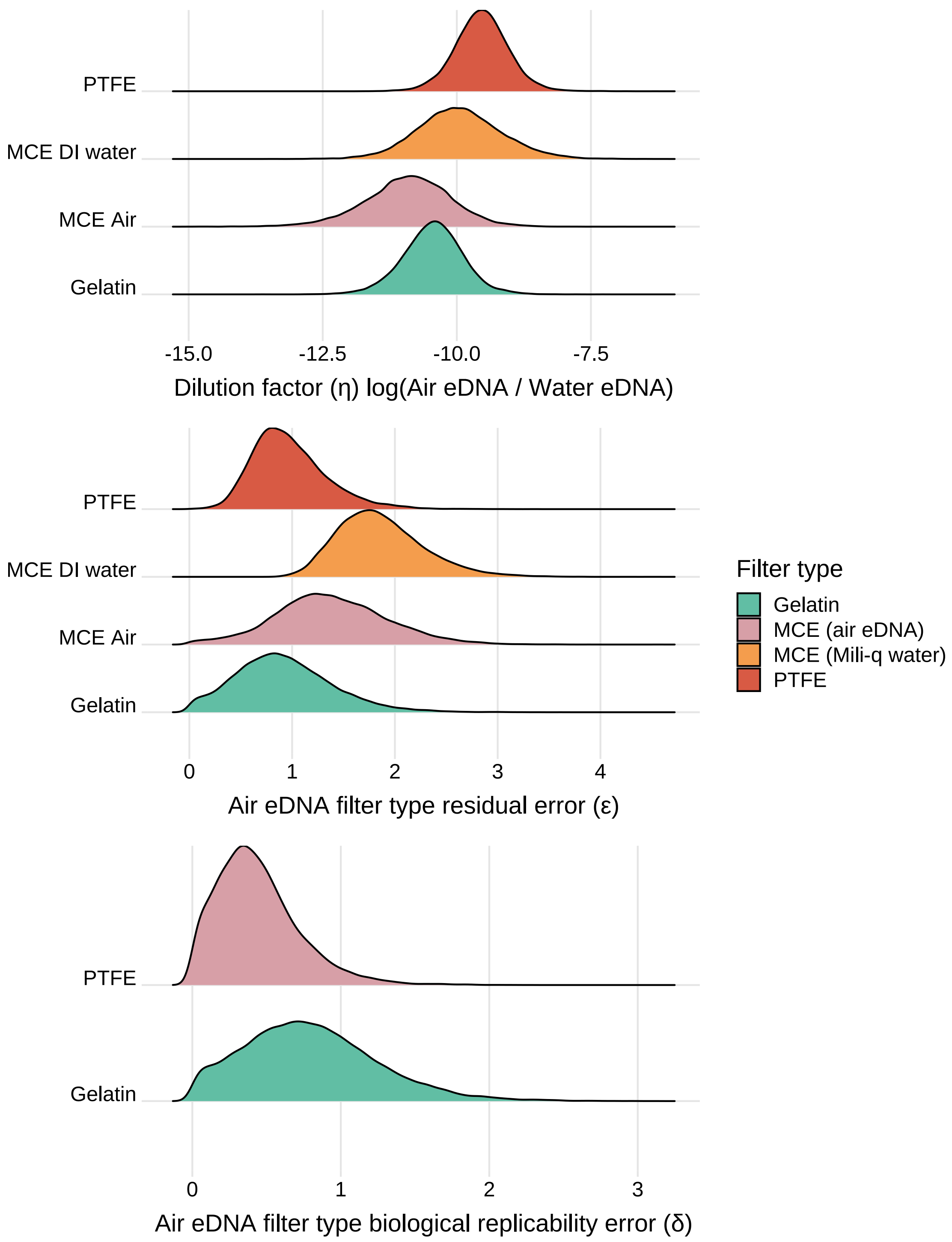

Figure S2: Density plots showing posterior distributions of: (A) water-to-air dilution factors (η), illustrating the magnitude of concentration reduction between aquatic and atmospheric matrices; (B) standard deviation (SD) of the residual error values (τ), representing the congruence (lower = better) between estimated eDNA concentrations between air and water; and (C) standard deviation (SD) of the biological replicability error (ρ), quantifying measurement consistency (lower = better) between technical replicates of two air filters (PTFE and gelatin filters). Each parameter is estimated for each of four different air eDNA collection methods (differentiated by colors).

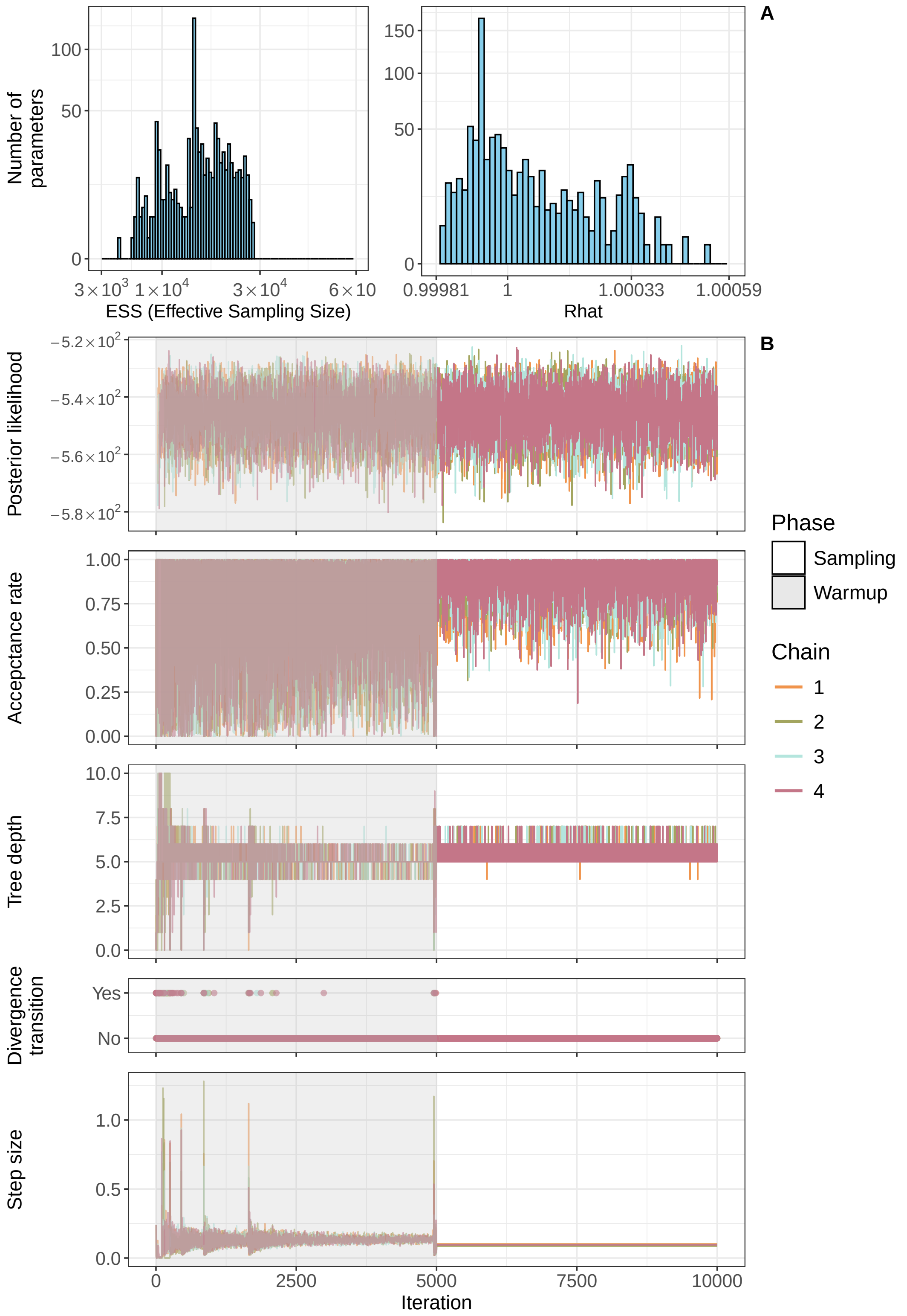

Figure S3: Bayesian model convergence diagnostics indicating (A) the distributions of effective sample size (ESS, left) and $\hat{R}$ values (right) for all parameters and (B) convergence metrics across iterations, including posterior likelihood, acceptance rate, tree depth, divergence transitions, and step size for four MCMC chains. Warmup and sampling phases are distinguished by background shading.

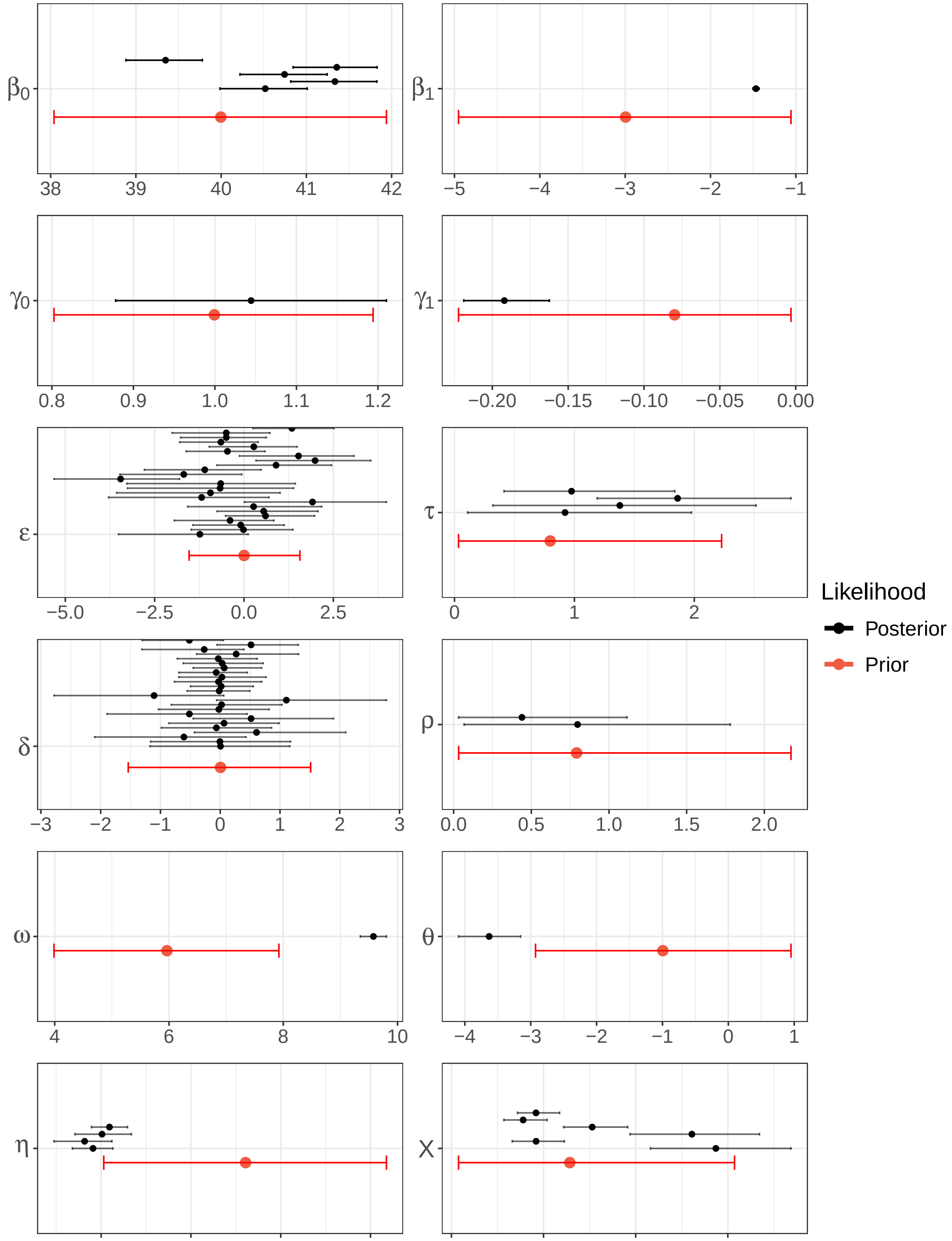

Figure S4: Prior sensitivity analysis for model parameters $(\beta_0, \beta_1, , \gamma_0, \gamma_1, \varepsilon, \tau, \delta, \rho, \omega, \theta, \eta, X)$. Black points and intervals represent posterior means and 95% confidence intervals respectively and red points and intervals show prior mean and 95% confidence intervals respectively.

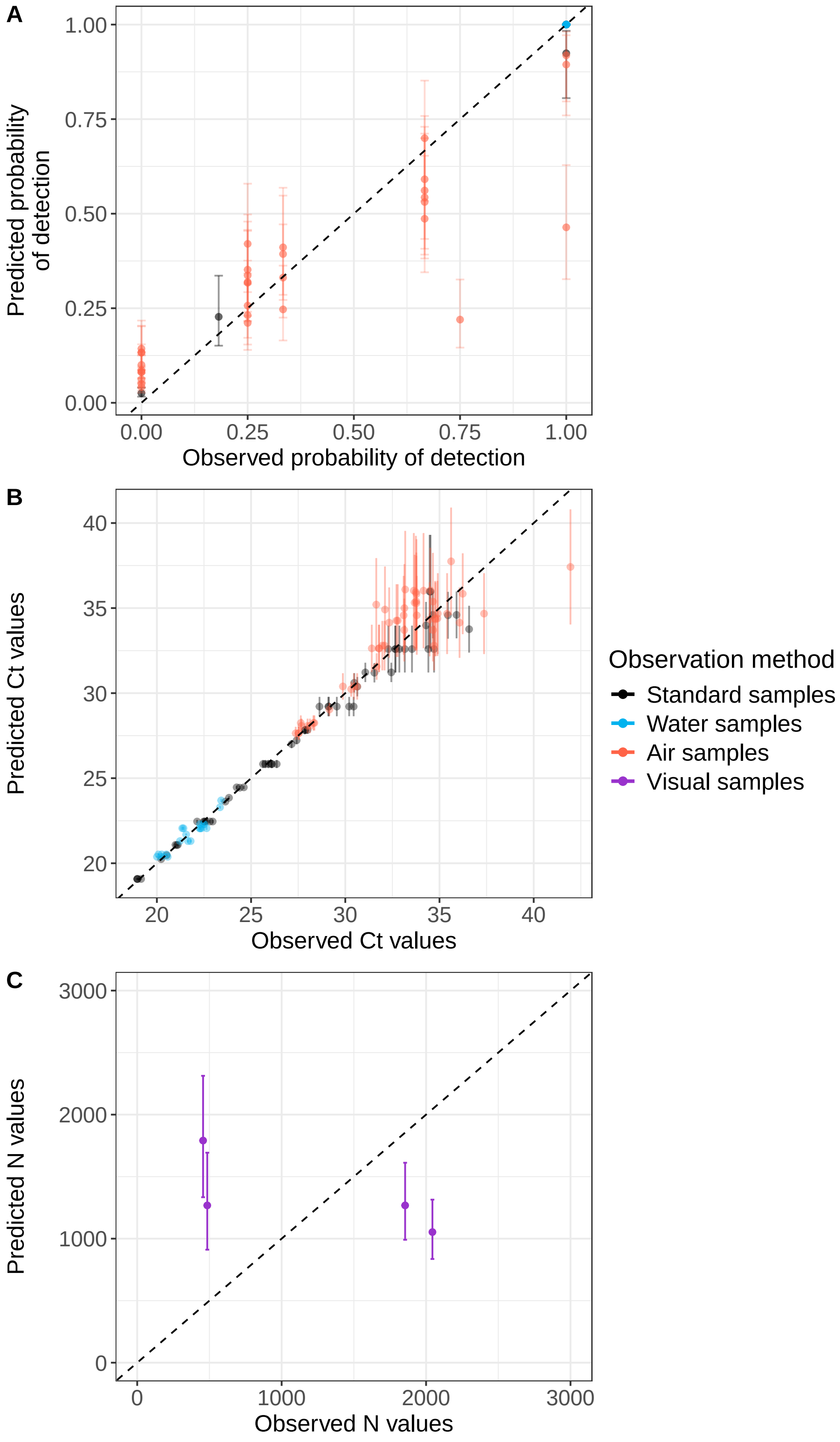

Figure S5: Posterior predictive checks comparing observed vs. model-predicted values for (A) detection probability of air and water samples alongside standard samples, (B) Ct values for air and water samples alongside standard, and (C) visual counts of fish (N).

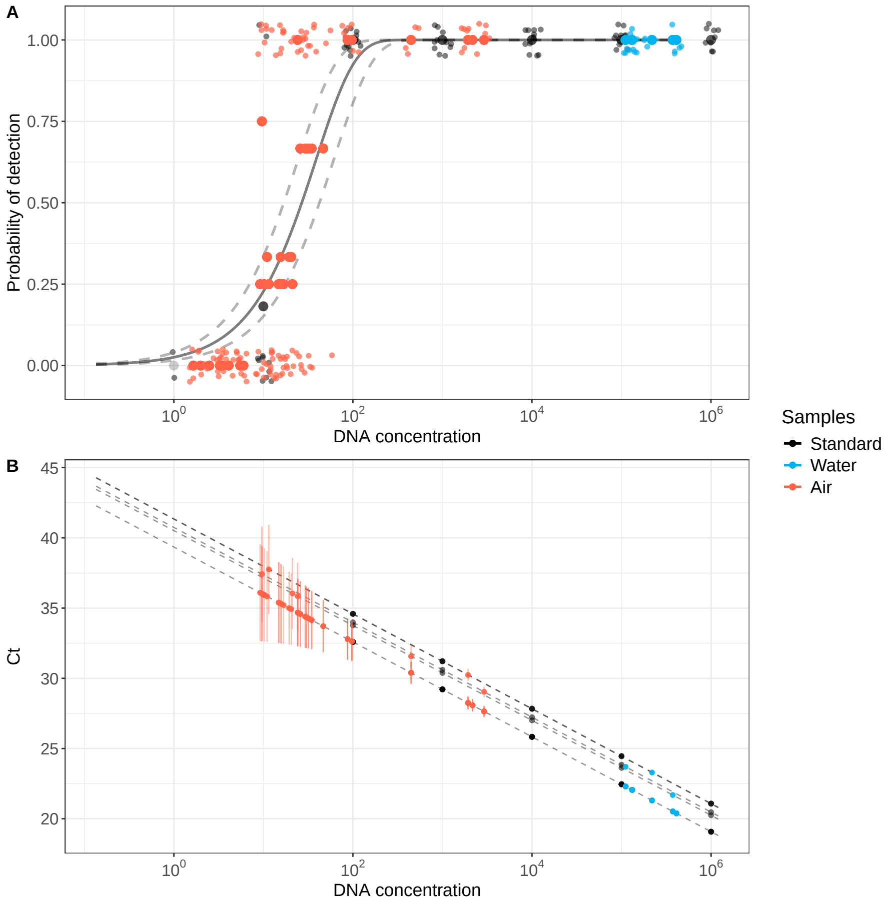

Figure S6: The probability of detection and Ct values along the eDNA concentration from the posterior estimates of the standard samples and water and air environmental samples indicating assay and qPCR machine performance.

	Description	Prior
	Data	
N	number of fish counted	-
E	elapsed days between counting	-
Z	qPCR amplification (yes=1; no=0)	-
Y	qPCR cycle threshold (Ct)	-
K	known DNA concentration (qPCR standards)	-
V	Reaction volume in μL	-
S	Surface area of air collection method cm/^{2}	-
F	Water volume filtered in the field in L	-
P	Passive air filter time deployment in days	-
	State processes	
X	unknown fish density in (fish $\cdot$ day^{-1})	$\mathcal{TN}(100, 100; 0, +\infty)$
W	unknown DNA concentration in water in (copies/L)	-
U	unknown eDNA concentration in water samples (copies/μL)	-
A	unknown DNA concentration in air in (copies/cm^2/day)	-
Q	unknown eDNA concentration in air samples (copies/μL)	-
	Transformed parameters	
λ	expected fish accumulated over E days	-
ψ	probability of positive qPCR amplification	-
μ	mean Ct values of qPCR	-
σ	standard deviation of qPCR Ct values	-
	Fixed parameters	
ϕ	overdispersion parameter of Negative Binomial	- ($\phi = 20$)
	Parameters	
ω	conversion parameter between fish density and DNA concentration	$\mathcal{N}(6, 1)$
η	dilution factor of DNA concentration from water to air	$\mathcal{N}(-2, 4)$
ε	time (t) specific error term (residual)	$\mathcal{N}(0, \tau)$
τ	standard deviation of residuals	$\mathcal{TN}(0, 1; 0, +\infty)$
δ	biological replicate error term (residual)	$\mathcal{N}(0, \rho)$
ρ	standard deviation of biological replicate error	$\mathcal{TN}(0, 1; 0, +\infty)$
θ	intercept of the qPCR probability of detection (ψ) relationship and eDNA concentration (K, W, and A)	$\mathcal{N}(-1, 1)$
$\beta0$	intercept of the linear relation between the mean Ct values (μ) and eDNA concentration (K, W, and A)	$\mathcal{N}(40, 1)$
$\beta1$	slope of the linear relation between the mean Ct values (μ) and eDNA concentration (K, W, and A))	$\mathcal{N}(-3, 1)$
$\gamma0$	intercept of the linear relation between the standard deviation of Ct values (σ) and eDNA concentration (K, W, and A)	$\mathcal{N}(1, 0.1)$
$\gamma1$	intercept of the linear relation between the standard deviation of Ct values (σ) and eDNA concentration (K, W, and A)	$\mathcal{N}(0, 0.1)$
	Index	
t	time (days)	-
j	filter type	-
b	biological sample replicate	-
p	qPCR plate	-
k	qPCR standard sample	-
r	qPCR technical replicate	-

Table S1: Data, state processes, parameters, transformed parameters, and subscripts employed in the joint Bayesian model and their prior distributions.

**Location**	**Date**	**Rain Yes/No**	**Precipitate (mm)**	**River water discharge (m)**
Issaquah Hatchery	2024-10-17	Yes	0.05	4.10
Issaquah Hatchery	2024-10-24	Yes	0.01	4.13
Issaquah Hatchery	2024-10-31	Yes	0.32	4.50
Issaquah Hatchery	2024-11-07	No	0	4.46
Issaquah Hatchery	2024-11-14	Yes	0.29	4.95
Issaquah Hatchery	2024-11-21	No	NA (sheltered bridge)	4.91

Table S2: Meteorological metadata collected from NOAA during the sampling events
